# The Effect of a Wordless, Animated, Social Media Video Intervention on COVID-19 Prevention: Online Randomized Controlled Trial

**DOI:** 10.2196/29060

**Published:** 2021-07-27

**Authors:** Alain Vandormael, Maya Adam, Merlin Greuel, Jennifer Gates, Caterina Favaretti, Violetta Hachaturyan, Till Bärnighausen

**Affiliations:** 1 Heidelberg Institute of Global Health Heidelberg University Heidelberg Germany; 2 Department of Pediatrics Stanford University School of Medicine Stanford, CA United States; 3 Icahn School of Medicine at Mount Sinai New York, NY United States; 4 Africa Health Research Institute Durban South Africa; 5 Department of Global Health and Population Harvard TH Chan School of Public Health Boston, MA United States

**Keywords:** social media, cultural and social implications, randomized controlled trial, list experiment, information literacy, COVID-19, pandemic, digital health, infodemiology, global health, public health

## Abstract

**Background:**

Innovative approaches to the dissemination of evidence-based COVID-19 health messages are urgently needed to counter social media misinformation about the pandemic. To this end, we designed a short, wordless, animated global health communication video (the CoVideo), which was rapidly distributed through social media channels to an international audience.

**Objective:**

The objectives of this study were to (1) establish the CoVideo’s effectiveness in improving COVID-19 prevention knowledge, and (2) establish the CoVideo’s effectiveness in increasing behavioral intent toward COVID-19 prevention.

**Methods:**

In May and June 2020, we enrolled 15,163 online participants from the United States, Mexico, the United Kingdom, Germany, and Spain. We randomized participants to (1) the CoVideo arm, (2) an attention placebo control (APC) arm, and (3) a do-nothing arm, and presented 18 knowledge questions about preventive COVID-19 behaviors, which was our first primary endpoint. To measure behavioral intent, our second primary endpoint, we randomized participants in each arm to five list experiments.

**Results:**

Globally, the video intervention was viewed 1.2 million times within the first 10 days of its release and more than 15 million times within the first 4 months. Knowledge in the CoVideo arm was significantly higher (mean 16.95, 95% CI 16.91-16.99) than in the do-nothing (mean 16.86, 95% CI 16.83-16.90; *P*<.001) arm. We observed high baseline levels of behavioral intent to perform many of the preventive behaviors featured in the video intervention. We were only able to detect a statistically significant impact of the CoVideo on one of the five preventive behaviors.

**Conclusions:**

Despite high baseline levels, the intervention was effective at boosting knowledge of COVID-19 prevention. We were only able to capture a measurable change in behavioral intent toward one of the five COVID-19 preventive behaviors examined in this study. The global reach of this health communication intervention and the high voluntary engagement of trial participants highlight several innovative features that could inform the design and dissemination of public health messages. Short, wordless, animated videos, distributed by health authorities via social media, may be an effective pathway for rapid global health communication during health crises.

**Trial Registration:**

German Clinical Trials Register DRKS00021582; https://tinyurl.com/6r4zkbbn

**International Registered Report Identifier (IRRID):**

RR2-10.1186/s13063-020-04942-7

## Introduction

Soon after the outbreak of the COVID-19 pandemic, health-related misinformation flooded the social media space [1,2]. Compelling, but often misleading, content captured the attention of a frightened global community [2]. The rapid and widespread dissemination of such misinformation on social media often overshadowed evidence-based recommendations released through more traditional public health communication channels. As a result, dangerous messages that increased the spread of COVID-19 and led to adverse health outcomes were allowed to spread to the estimated 3.8 billion people worldwide who use social media [3]. Tedros Ghebreyesus, Director-General of the World Health Organization warned, “We’re not just fighting an epidemic; we’re fighting an infodemic” [2].

There is a critical need to rapidly disseminate evidence-based informational videos on social media channels to counteract the epidemic of COVID-19 misinformation. To date, public health efforts have focused on correcting misinformation and debunking myths [4]. As such, these measures have almost exclusively been reactive rather than proactive. The corrective content itself has not been designed to incorporate the very characteristics that support the viral spread of content on social media [5]. For this reason, social media interventions designed to correct misinformation have unfortunately demonstrated far less impact than the content they aim to correct [4]. Researchers studying this emerging global health communication approach have urged health authorities to enter the social media arena more intentionally, with the aim of disseminating valid information, evaluating its impact, and reducing the knowledge translation gap [3]. Social media health messaging interventions need to do more than convey reliable information. They must be as emotionally compelling as they are evidence-based, if public health authorities are to reach broad, global audiences [5]. They also need to be accessible and tailored for cross-cultural acceptability [6].

In March 2020, we designed a short, wordless, and animated video to disseminate information about preventing the spread of COVID-19 [7,8]. The intervention video (the CoVideo) promotes evidence-based messages that focus on a set of preventive behaviors such as hand washing, social distancing, and the sanitation of kitchen surfaces, among others. Importantly, the CoVideo incorporates audience engagement characteristics that motivate widespread sharing on social media [5]. For example, it includes a compelling, familiar narrative and characters that are culturally agnostic; and the soundtrack is designed to evoke high-arousal emotions [9], which reflects the anxiety, altruism, and solidarity [10] of the global community. The CoVideo was released on Stanford Medicine’s YouTube channel on March 21, 2020, and within 10 days reached 332,000 views on YouTube, 220,000 views on Instagram, 294,000 views on Facebook, and 402,000 views on Twitter, with a cumulative count of 1.2 million [6]. It continued to spread organically across social media channels, due to reposting by several global health authorities, including government departments of health, community health organizations, and media channels around the world [6]. Within 4 months, the CoVideo had reached more than 5.8 million people through their social media accounts.

In this study, we evaluate the effectiveness of the CoVideo to improve knowledge and behavioral intent toward COVID-19 prevention. According to the Theory of Planned Behavior, the intention to act is considered to be the immediate determinant of action [11]. Here, we frame behavioral intent as representing the participant’s commitment to undertake COVID-19 prevention behaviors in the next 7 days, which is the second outcome of our study [12,13]. As the primary outcome of our study, we aim to measure changes in knowledge about COVID-19 prevention. Knowledge is often considered to be a necessary but not sufficient condition for motivating a healthy behavior [14]. Specifically, the Theory of Planned Behavior posits that knowledge is more likely to be correlated with behavior if correct answers on the knowledge test support the practice of that behavior [15]. Results from this study, which incorporates several innovations in global health communication, can inform the development of future videos to disseminate evidence-based recommendations related to COVID-19 and other public health emergencies.

## Methods

### Trial Design

This is a multisite, parallel-group randomized controlled trial (RCT) comparing the effectiveness of a short informational video on COVID-19 prevention. To evaluate the effectiveness of the CoVideo, we enrolled participants from five countries into a large, online RCT. We randomly assigned participants to the CoVideo [7], an attention placebo control (APC) video [16], or no video (do-nothing arm), and measured change in knowledge of COVID-19 prevention behaviors (first endpoint) and change in self-reported behavioral intent toward COVID-19 prevention (second endpoint). Our RCT included two innovative experimental approaches. First, we used the APC to isolate the content effect of the CoVideo (the active component of the COVID-19–related health messaging and its delivery design) from the attention effect of watching a video (the inactive component of the intervention). Second, we nested a list experiment in each trial arm to reduce socially desirable responses to the behavioral intent questions. Both approaches were leveraged to improve the accuracy of our estimates. The study and its outcomes were registered with the German Clinical Trials Register [17] on May 12, 2020 (DRKS00021582). Ethical approval was obtained from the Stanford University Institutional Review Board on April 12, 2020 (#55820). There were no changes to the trial outcomes or methods after the trial commenced.

### Participants

We used an online platform called Prolific [18] to enroll participants from the United States, Mexico, the United Kingdom, Germany, and Spain into the RCT [8]. Participant eligibility included being 18 to 59 years of age (male, female, or other), being a resident of one of the five countries, and having proficiency in English, German, or Spanish. The trial was hosted and deployed on Gorilla [19], which is a cloud platform that provides versatile tools to undertake online, experimental, and behavioral research [20]. Participants were compensated an equivalent of £1 (US$ 1.39) for a 10-minute completion time. To prevent duplicate participation, Prolific uses a number of tracking mechanisms, including IP and internet service provider address detection [21].

### Procedures

Participants began by answering basic demographic questions about their age, sex, primary language, country of residence, and highest education completed.

The Gorilla algorithm then randomly assigned participants 1:1:1 to the CoVideo, APC video, or do-nothing groups. Participants were required to watch the CoVideo or the APC video once from start to finish. The CoVideo is animated with sound effects but does not include any words, speech, or text. It explains how the novel coronavirus is spread (airborne, physical contact) and recommends best practices to prevent onward transmission (staying at home, not congregating in public spaces, and sanitizing hands/surfaces). It also covers the mass media coverage of the outbreak and the public’s response to this media coverage, which includes a subplot on the stockpiling of essential goods, and the impact thereof on health care services and resources (eg, doctors being unable to access protective equipment). The total duration of the CoVideo is 2 minutes, 30 seconds.

The APC is also a wordless, animated video with the same duration as the CoVideo. Its content describes how small choices become actions, which become habits, which become a way of life. We included an APC to account for possible attention effects elicited by the video format. APC conditions should mimic the “inactive” components of an intervention—the effect of watching the video—while not containing any of the “active” intervention components—the content delivered by the video [22]. We did not make the assumption that the CoVideo is better than nothing (ie, no video). It is possible that the CoVideo could motivate reactance to our COVID-19 prevention message [23-25].

After completing the intervention (CoVideo, APC, do-nothing), participants answered 18 knowledge questions on preventive COVID-19 behaviors. All items required true or false responses, and all participants received the knowledge items. After completing the knowledge questions, participants then completed five list experiments. For each list experiment, we randomized participants 1:1 to a control list or a control list plus a sensitive item about behavioral intent toward social distancing, washing hands, cleaning dishes, cleaning kitchen surfaces, and the stockpiling of essential goods. The control group received a list of 5 items that were unrelated to COVID-19. For example, in the first list experiment, we asked: “How many of the five statements do you agree with? We don’t want to know which ones, just answer how many: 1. Spend time watching TV, 2. Do the vacuuming, 3. Pick a fight with my partner, 4. Eat a low sugar diet, 5. Rinse my nose with salt water daily.” The treatment group received the same 5 items and 1 additional “sensitive” item, “Go out with my friends,” which indicates behavioral intent to social distance (or not) during lockdown restrictions. We used the list experiments to reduce social desirability bias [26,27] and designed them in line with best practices [28].

### Statistical Analysis

We summarized the participant characteristics by obtaining mean (SD) values for age, gender, primary language, country of residence, and education status. Using the Gorilla platform, we identified and excluded participants from the analysis who were lost, defined as those who did not complete the survey from start to finish. Because we could not determine if participants watched some or all of the CoVideo or APC video, we used an intention-to-treat analysis.

For the first endpoint, we calculated a knowledge score for each participant by adding the correct responses (min=0, max=18). Participants had a time limit of 30 seconds to answer each knowledge item, preventing them from searching for answers on the internet. If the participant timed out, they received a missing value of 9. This missing value was recoded as an incorrect answer to the knowledge item, since the participant could not correctly answer the question in the allotted time. We used an analysis of variance (ANOVA) model with and the Tukey honestly significant difference to test for statistically significant differences (with α=.05) in mean knowledge between the CoVideo, APC, and do-nothing arms. The ANOVA model is *y*=*b*_1_VideoArm, where *y* is the number of knowledge statements that the participant correctly answered and VideoArm represents the treatment arm.

For the second endpoint, we calculated the prevalence of behavioral intent to perform COVID-19 preventive behaviors for each list experiment. Let *C_j_* denote the number of items that the *j*th participant selected from the control list (min=0, max=5), and let *T_j_* be the number of items that the *j*th participant selected from the treatment list (min=0, max=6). We calculated the mean score for the control list, denoted by 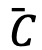 and treatment list, denoted by 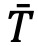, for the *i*th list experiment (*i*=1,…,5). Let the superscripts *cov* denote the CoVideo, *apc* denote the APC, and *no* denote the do-nothing arms, and let *k* denote the *k*th trial arm (*k* ∈ [*cov, apc, no*]). For list experiment *i* and trial arm *k*, we then estimated the prevalence of behavioral intent, denoted by *P_i_^k^*, as the difference between the treatment and control, such that *P_i_^k^*= (*T_i_^k^ – C_i_^k^*) × 100. From these estimates, we calculated the total, content, and attention effect of the CoVideo. Let *D_i_^Tot^* denote the total effect, which is estimated by *P_i_^cov^* – *P_i_^no^*; and let *D_i_^Att^* denote the attention effect, which is estimated by *P_i_^apc^* – *P_i_^no^*. These analyses are analogous to difference-in-difference analyses, which we implemented by specifying the main and interaction terms in an ordinary least squares (OLS) regression model. The OLS equation for the *i*th list experiment is given as:

*y* = *b*_0_ + *b*_1_VideoArm + *b*_2_TreatList + *b*_3_(VideoArm × TreatList),

where *y* is the number of statements in the list that the participant agreed with, VideoArm indicates the *k*th arm, and TreatList indicates assignment to the treatment or control list. We calculated standard errors, 95% CIs, and *P* values (with α=.05) for linear combinations of coefficients from the OLS model.

### Informed Consent

All participants underwent a process of informed consent on the Prolific platform. The consent form explained the purpose of the study, the risks and benefits of the research, and how to contact the study investigators (or the Stanford University ethics review board). By clicking the link, participants agreed to participate in our study, and were redirected to the Gorilla platform, where additional information was given. Participants could withdraw from the study at recruitment or at any point during the experiment.

### Confidentiality

Each participant was assigned a unique, anonymized ID on Prolific and had no identifying information associated with it. We informed participants that their names could be revealed to us if they emailed the study investigators. The study investigators kept this information confidential.

### Blinding

Because Prolific handled the interaction between the study investigators and participants, the participants were completely anonymous to the study investigators. Participants self-responded to the survey questions and self-submitted their responses anonymously on the Gorilla platform. Only the participant’s unique, anonymized ID was used to manage the linking between the Prolific and Gorilla platforms. The study investigators were blinded to the group allocation [8].

### Adverse Event Reporting and Harms

No adverse events or harms were observed given the online format of the trial.

### Data Availability

The data that support the findings of this study are available from the corresponding author upon request.

## Results

**Figure 1:**
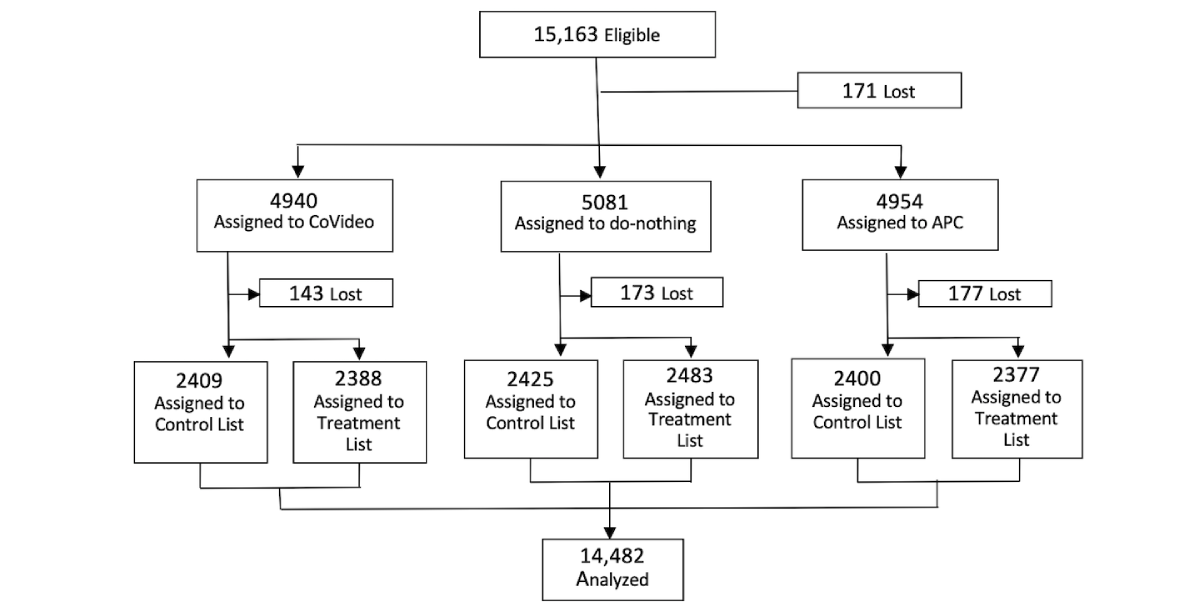
Trial design. After recruitment, participants were randomly assigned (1:1:1) to the CoVideo, attention placebo control (APC), or do-nothing arms. Participants in each trial arm were also randomized (1:1) to a control list (5 items; no sensitive item) or a treatment list (6 items; with 1 sensitive item) about behavioral intent toward social distancing, washing hands, cleaning dishes, cleaning kitchen surfaces, and the stockpiling of essential goods.

Between May 13, 2020, and June 23, 2020, 15,163 participants from the United States, Mexico, the United Kingdom, Germany, and Spain were enrolled in our RCT. Between recruitment and randomization, 171 participants were lost and 14,992 participants were randomly assigned to the CoVideo (n=4940), APC (n=4954), and do-nothing (n=5081) arms (Figure 1). After randomization, another 173 (do-nothing), 177 (APC), and 143 (CoVideo) participants were lost for unknown reasons (possibly due technical issues like lost internet connection; difficulties linking to the video host, YouTube; server complications, etc). A total of 14,482 participants completed the trial and contributed data to the final analysis.

The majority of participants reported their residence in the United Kingdom (n=8519, 58.8%) or the United States (n=3765, 26%), and 84.9% (n=12,288) of participants reported English as their first language. The sample was relatively well educated, with 81.6% (n=11,812) having some college education or higher (bachelor’s, master’s/equivalent, or PhD). Table 1 shows the percentage of participants in each arm and treatment list by age, gender, country of residence, educational status, and primary language.

**Table 1:** Baseline demographic characteristics of participants by trial and list experiment arms (collected from 14,482 participants between May 2020 and June 2020).

Characteristic			Do-nothing			APC^a^			CoVideo			*P* value	
		Control list, n (%)		Treatment list, n (%)	Control list, n (%)		Treatment list, n (%)	Control list, n (%)		Treatment list, n (%)			
**Age**													.98
	18-24 years	672 (27.7)		691 (27.8)	649 (27.0)		640 (26.9)	656 (27.2)		667 (27.9)			
	25-34 years	877 (36.2)		902 (36.3)	866 (36.1)		880 (37.0)	884 (36.7)		848 (35.5)			
	35-44 years	475 (19.6)		502 (20.2)	484 (20.2)		456 (19.2)	479 (19.9)		470 (19.7)			
	45-54 years	285 (11.8)		295 (11.9)	297 (12.4)		279 (11.7)	280 (11.6)		299 (12.5)			
	55-59 years	116 (4.8)		93 (3.7)	104 (4.3)		122 (5.1)	110 (4.6)		104 (4.4)			
**Gender**													.38
	Female	1316 (54.3)		1298 (52.3)	1353 (56.4)		1269 (53.4)	1306 (54.2)		1310 (54.9)			
	Male	1090 (44.9)		1167 (47.0)	1037 (43.2)		1092 (45.9)	1088 (45.2)		1063 (44.5)			
	Other	19 (0.8)		18 (0.7)	10 (0.4)		16 (0.7)	15 (0.6)		15 (0.6)			
**Country of residence**													>.99
	Germany	118 (4.9)		135 (5.4)	132 (5.5)		116 (4.9)	130 (5.4)		124 (5.2)			
	Mexico	116 (4.8)		119 (4.8)	119 (5.0)		117 (4.9)	114 (4.7)		117 (4.9)			
	Spain	124 (5.1)		126 (5.1)	125 (5.2)		121 (5.1)	123 (5.1)		122 (5.1)			
	United Kingdom	1418 (58.5)		1453 (58.5)	1384 (57.7)		1437 (60.5)	1429 (59.3)		1398 (58.5)			
	United States	649 (26.8)		650 (26.2)	640 (26.7)		586 (24.7)	613 (25.4)		627 (26.3)			
**Education status**													.35
	Primary school	66 (2.7)		90 (3.6)	66 (2.8)		61 (2.6)	83 (3.4)		87 (3.6)			
	High school	360 (14.8)		377 (15.2)	383 (16.0)		360 (15.1)	364 (15.1)		373 (15.6)			
	Bachelor’s, some college	1551 (64.0)		1570 (63.2)	1529 (63.7)		1507 (63.4)	1526 (63.3)		1497 (62.7)			
	Master’s/PhD	448 (18.5)		446 (18.0)	422 (17.6)		449 (18.9)	436 (18.1)		431 (18.0)			
**First language**													>.99
	German	117 (4.8)		135 (5.4)	130 (5.4)		116 (4.9)	128 (5.3)		124 (5.2)			
	English	2068 (85.3)		2103 (84.7)	2026 (84.4)		2022 (85.1)	2044 (84.8)		2025 (84.8)			
	Spanish (Mexico)	124 (5.1)		126 (5.1)	125 (5.2)		123 (5.2)	123 (5.1)		122 (5.1)			
	Spanish	116 (4.8)		119 (4.8)	119 (5.0)		116 (4.9)	114 (4.7)		117 (4.9)			

**Figure 2:**
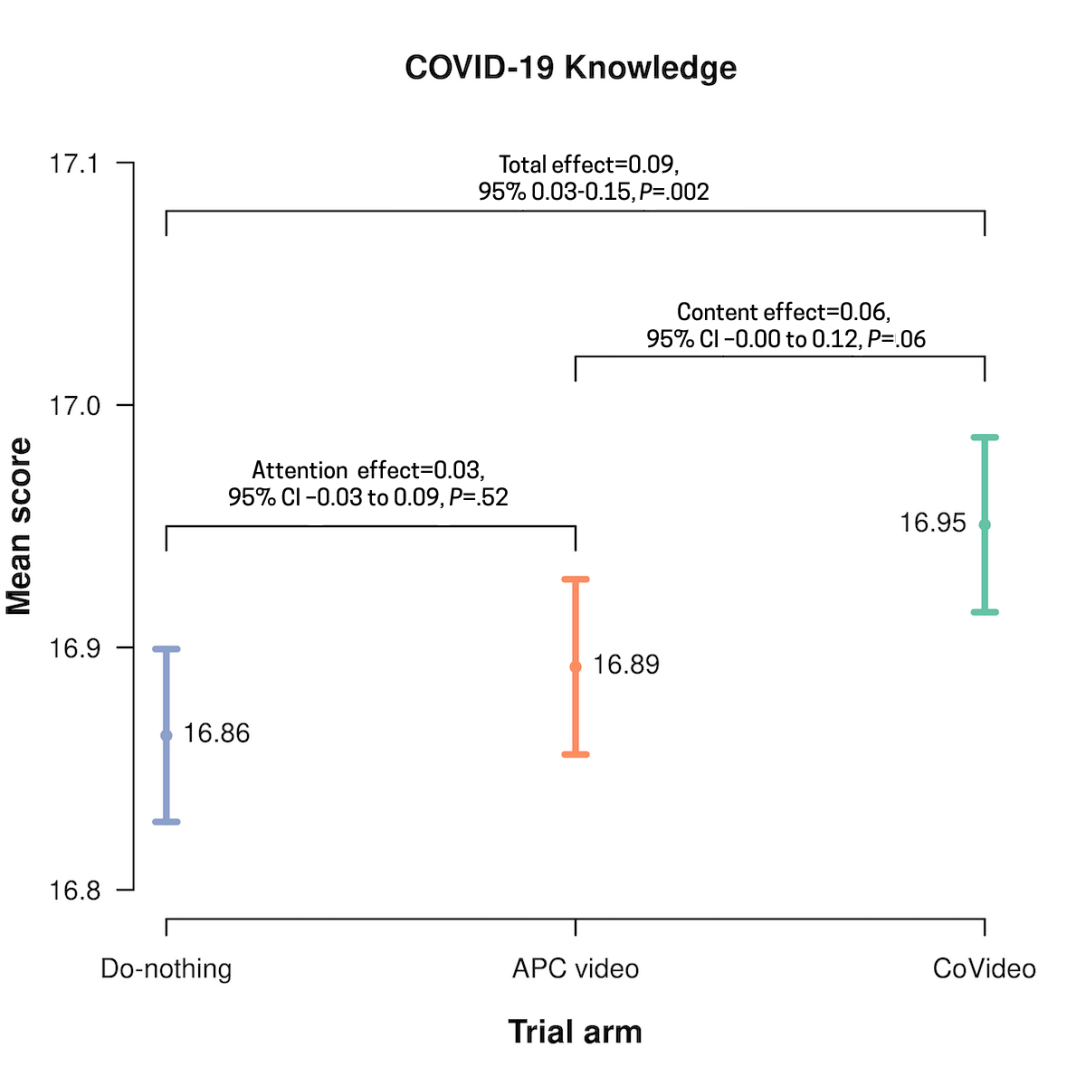
Mean scores for the COVID-19 knowledge questions by trial arm (N=14,482). Differences between the CoVideo, attention placebo control (APC), and do-nothing arms are reported with *P* values. Total effect represents the difference in means between the CoVideo and do-nothing arms, attention effect represents the difference in means between the APC and do-nothing arms, and content effect represents the difference in means between the CoVideo and APC arms.

**Figure 3:**
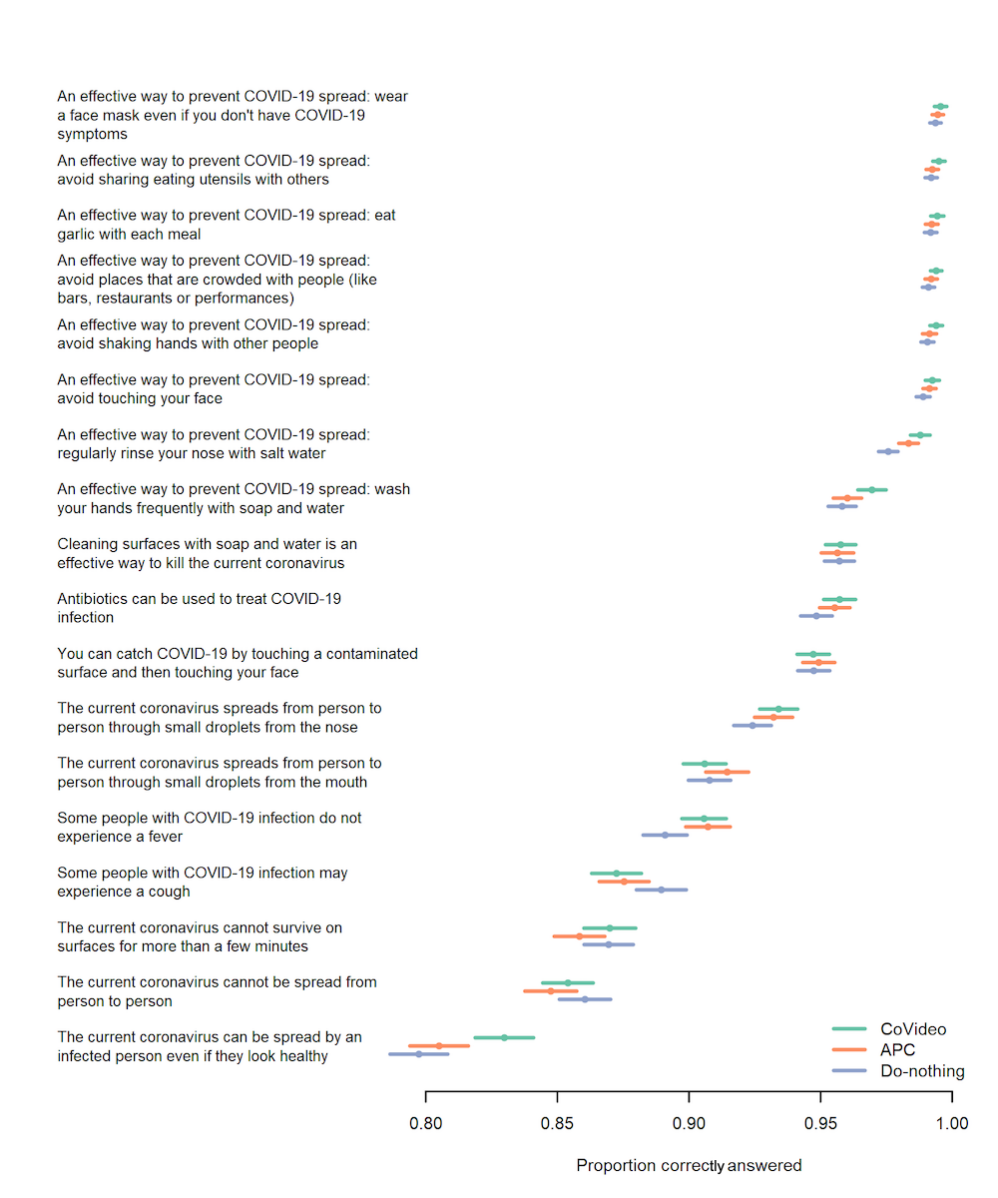
The proportion of correct answers for each knowledge item in the CoVideo, attention placebo control (APC), and do-nothing arms (N=14,482).

The knowledge questionnaire had an acceptable reliability correlation coefficient of 0.65 (split-half). Overall, there was extraordinarily high attainment of COVID-19 knowledge. In the do-nothing arm, participants correctly answered 16.86 (95% CI 16.83-16.90) out of 18 items, which is a 93.7% correct response rate (Figure 2). With this high baseline score, the CoVideo could therefore only increase knowledge by a maximum of 1.14 points. Relative to the do-nothing arm, the CoVideo increased knowledge by 0.09 points (mean 16.95, 95% CI 16.91-16.99; *P*=.002), which represents an increase of 7.6% (0.09/1.14) (Figure 2). The average score for the APC arm was 16.89 (95% CI 16.86-16.93), a correct response rate of 93.8%. When we removed the attention effect of the video format, the CoVideo increased overall knowledge by 0.06 points (*P*=.06), which represents an increase of 5.3% (0.06/1.11). Figure 3 shows the proportion of correct responses to each of the 18 knowledge items (see also Table S1 in Multimedia Appendix 1). The highest correctly answered item (“An effective way to prevent COVID-19 spread is to wash your hands frequently with soap and water”) had a correct response rate of 99.4%; most items had a >90% correct response rate.

**Figure 4:**
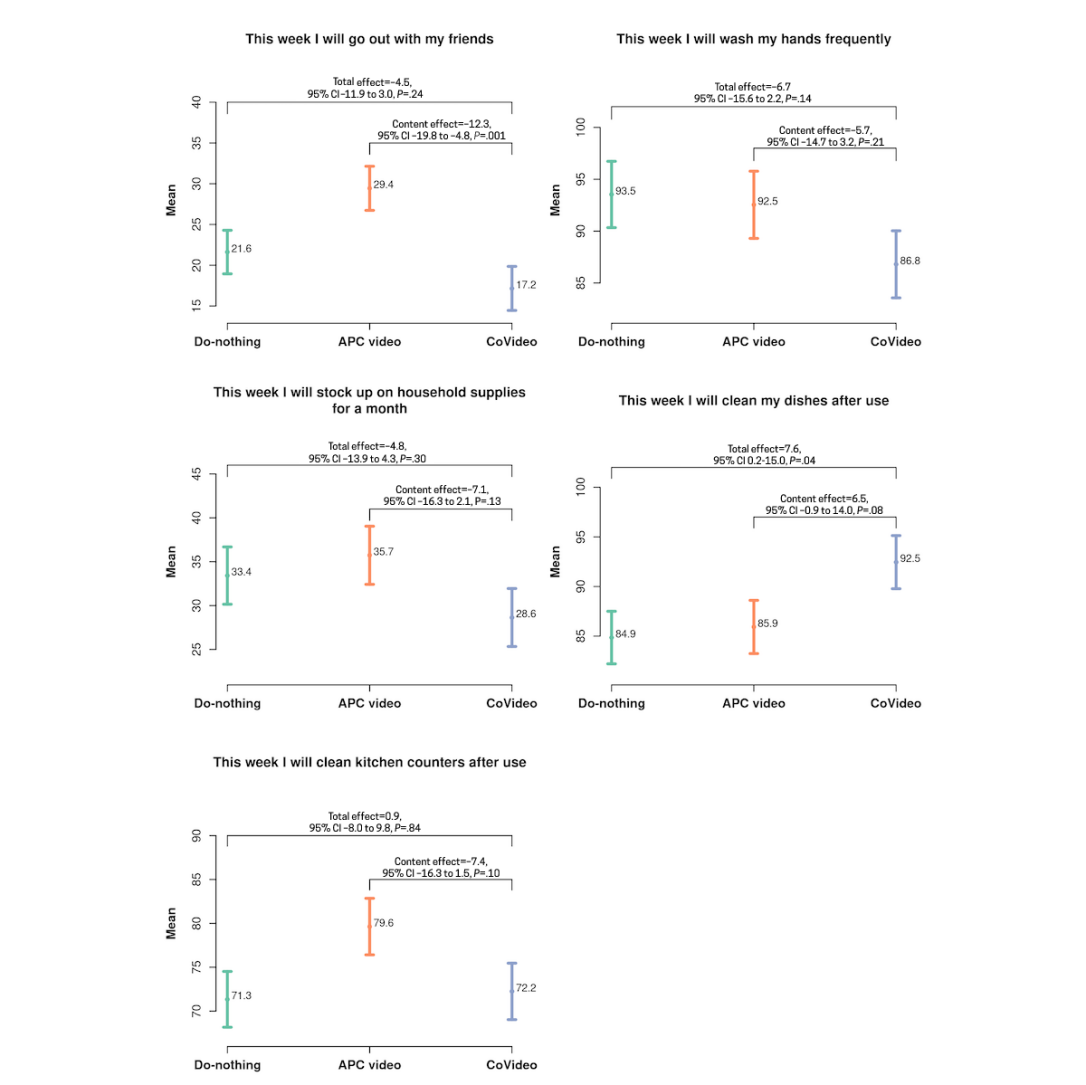
The prevalence of behavioral intent for each of the five list experiments with 95% CIs. Differences between the CoVideo, attention placebo control (APC), and do-nothing arms are reported with *P* values. Total effect represents the difference in means between the CoVideo and do-nothing arms, attention effect represents the difference in means between the APC and do-nothing arms, and content effect represents the difference in means between the CoVideo and APC arms.

Figure S1 in Multimedia Appendix 1 shows the mean scores for the five list experiments by trial arm and list group. These mean scores were used to calculate the prevalence of behavioral intent for each preventive COVID-19 behavior, including the total and content effects with 95% CIs and *P* values (Figure 4). Scores for the treatment list are higher because the treatment list has 6 items and the control list has 5 items. For a given trial arm, the difference between the treatment and control means represents the prevalence of intent to undertake the preventive COVID-19 behavior. For example, for the first list experiment in the CoVideo arm (“this week I will go out with friends”), 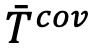
=2.20 is the treatment mean and 
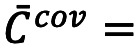
 2.03 is the control mean. The prevalence is then 
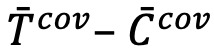
 × 100 = (2.20–2.03) × 100 = 17.2, as shown in Figure 2. Similarly, the prevalence for the APC arm is 
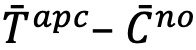
 × 100 = (2.33–2.03) × 100 = 29.4. For our secondary outcome, we report that participants in the CoVideo arm had lower behavioral intent to go out with friends when compared with the APC (content effect=–0.123, *P*<.001) and do-nothing (total effect=–0.045, *P*=.24) arms.

## Discussion

In this study, we tested an intervention with several innovations in global health communication that catalyzed a broad, organic global reach on social media [6,8-10]. The intervention, called the CoVideo, packaged critical health messages about COVID-19 prevention within a compelling, familiar narrative, using characters that were free of cultural identifiers and a soundtrack designed to evoke high-arousal emotions. Our results showed that baseline levels of COVID-19 prevention were high, and that the CoVideo intervention increased this prevention knowledge by another 7.6% and 5.3% relative to the do-nothing and APC arms, respectively. It was also found that the CoVideo intervention improved behavioral intent toward COVID-19 prevention when compared with the APC and do-nothing arms.

To evaluate the effectiveness of the CoVideo on knowledge and behavioral intent toward COVID-19 prevention, we used a large, online RCT to enroll 15,163 participants from the United States, Spain, Germany, the United Kingdom, and Mexico. The results for our first endpoint showed high knowledge of COVID-19 prevention behaviors across the five countries. For the three trial arms, the average number of correct answers was nearly 17 out of 18 items, a correct response rate of approximately 94%. Moderate to high knowledge levels about COVID-19 prevention measures among the general public were also observed earlier in Ecuador [29] and the United States [30-32]. On the other hand, a recent systematic review on knowledge, attitude, and practices toward the COVID-19 pandemic on the American continent concluded that many people have insufficient knowledge about the virus, highlighting the need to develop effective educational tools and materials on COVID-19 prevention [33]. The high baseline levels of COVID-19 knowledge in our study could be due to the delay of several weeks that occurred between the original release of the CoVideo and the launch of our online trial, as we awaited ethics approval, and designed and registered the trial. This lag likely facilitated exposure of our participants to COVID-19 prevention messages from other sources. Our results suggest, as we drift deeper into the pandemic, it may be unnecessary to spend more money on public health campaigns to improve COVID-19 prevention knowledge in the five countries from which we enrolled participants.

An important study finding was that the CoVideo improved already high levels of COVID-19 prevention knowledge. In the do-nothing and APC arms, only 1.14 and 1.11 additional correct items were needed to reach a perfect (100%) score, respectively. Our results showed that the CoVideo boosted COVID-19 prevention knowledge by another 7.6% relative to the do-nothing ceiling and by 5.3% relative to the APC ceiling. It seems plausible, therefore, that the CoVideo could significantly improve COVID-19 prevention knowledge in countries where baseline knowledge levels are currently low or moderate.

For our second endpoint, we nested a list experiment in each trial arm to evaluate the effect of the CoVideo on self-reported behavioral intent toward COVID-19 prevention. We used this experimental approach because it is likely that participants (at the time of enrollment) were already primed to give socially desirable responses to questions about COVID-19 prevention. The indirect questions (ie, how many statements do you agree with) provide protection to participants who have no behavioral intent toward COVID-19 prevention, without revealing this intention directly [27]. Our results showed that behavioral intent to go out with friends during stay-at-home recommendations and to stockpile household goods was lower in the CoVideo arm when compared with the APC and do-nothing arms, but not significantly so. We also observed that participants had higher behavioral intent to prevent COVID-19 spread by cleaning dishes after use when compared with the do-nothing arm (significantly different) and APC arm (not significantly different). Several studies have used the list experiment technique in the context of COVID-19 and found that list experiments were less favorable than simpler, traditional measurements, concluding that social desirability had no impact on the reported compliance with COVID-19 regulations [34,35]. On the contrary, other scholars have argued that the list experiment approach counters social desirability and is, therefore, less likely to introduce measurement errors presented by direct questions that measure self-reported compliance with COVID-19 guidance [36,37].

Our study is innovative in its use of both a list experiment and an APC video. Our APC video was selected to account for the possible attention effects elicited by the CoVideo intervention. The APC was designed to mimic the inactive components of the CoVideo intervention (the effect of watching a video of the same length), while not containing the active intervention component (the content of the COVID-19–related health messaging and its delivery design) [22]. The APC, therefore, enabled us to decompose the total intervention effect, which is the difference in knowledge means between the CoVideo and do-nothing groups, into the sum of the content and attention effects. We are not aware of any study that has used this approach to isolate the active component (the content effect) of the intervention video itself. For this purpose, we advise researchers using APCs to choose their APC topic carefully, and to avoid any potential effect of the placebo content on the outcomes being studied.

Our study had several limitations. At the time of our study, no validated scale on COVID-19 knowledge prevention existed. Nevertheless, we used best practices from the survey methods field to inform the design and development of the knowledge questions [38]. Another limitation is that we could not determine if participants watched some or all of the CoVideo or APC video. Once participants were randomized to a video, they could not skip to the end or fast-forward without ending the study. However, it is possible in some cases that the participants could have been engaged in other activities while the video was playing. Because of potential noncompliance, we used an intention-to-treat analysis. One possible limitation is that high baseline knowledge likely reflects the high educational status of our online sample, with 81.6% having some college education or higher (bachelor’s/equivalent, master’s, PhD). Our sample was likely more educated than the general populations of the United States, the United Kingdom, Germany, Spain, and Mexico. A similar educational distribution has been reported in a recent web-based study on COVID-19 knowledge in the United States and United Kingdom [39].

Together, the findings of this study present innovative propositions for content design, dissemination, and evaluation of rapid global health communication interventions. Content designs that emphasize cultural accessibility, convey a compelling narrative, and elicit high-arousal emotions could fuel rapid dissemination across the 3.8 billion global citizens currently using social media. The wordless, animated approach also minimizes barriers traditionally associated with underlying differences in language and literacy levels. Given the massive global penetration of social media, short, animated, wordless video messages, designed to spread organically, may help public health authorities reach people where they are (ie, social media). Evaluating these interventions using online trials, APCs and list experiments can help expedite results and strengthen our efficacy evaluations. The value of such an approach becomes especially apparent during global crises in which lost weeks translate into lost lives. Accessible and compelling video health messages that lean on the shared characteristics of our global community could facilitate the spread of time-sensitive health messages. Public health authorities poised to implement these innovative health communication solutions could better support a global community facing unprecedented, shared challenges.

### Authors’ Contributors

AV, MA, and VH wrote the paper. AV and CF undertook the statistical analysis. MA designed, produced, and created the CoVideo. TB, AV, and MA designed the trial. AV, TB, MA, and MG contributed to the questionnaire development. All authors provided comments and feedback.

## Appendix

Multimedia Appendix 1 Supplementary material.

Multimedia Appendix 2 CONSORT-EHEALTH checklist (V 1.6.1).

## Abbreviations

ANOVA: analysis of variance
APC: attention placebo control
OLS: ordinary least squares
RCT: randomized controlled trial
