# Supplementary material for: The Effect of a Wordless, Animated, Social Media Video Intervention on COVID-19 Prevention: Online Randomized Controlled Trial
Source: JMIR Public Health Surveill. 2021 Jul 27;7(7):e29060. doi: 10.2196/29060 (PMC8317990; doi:10.2196/29060)
Supplement: Multimedia Appendix 1 [file publichealth_v7i7e29060_app1.docx]

**Supplement**

## **Sample size**

We calculated the sample size needed for pairwise comparisons between three groups using a one-way analysis of variance (ANOVA). The formula to calculate the sample size is [1]:


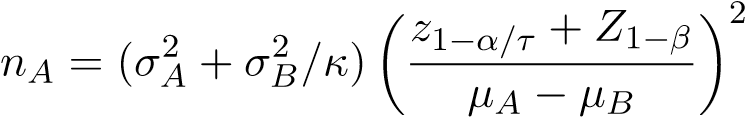


where *κ* = 1, which is the matching ratio, *µ_A_* and *µ_B_* are the group A and B means, *σ_A_* and *σ_B_* are the group A and B standard deviations, *α* = 0*.*05 is the Type-I error, *β* = 0*.*20 is the Type-II error, *z* is the quantile function, and *τ* = 2 is the number of comparisons to be made. To detect a small difference of 0.1 between the knowledge scores of two trial arms, we assumed a mean of *µ_A_* = 14*.*0 and *µ_B_* = 14*.*1 and *σ_A_* = 1*.*5 and *σ_B_* = 1*.*5. We select *µ* on the expectation that participants will get 14 out of 18 knowledge items correct. This gives a sample size of *n_A_* = *n_B_* = *n_C_* = 3*,*532, so *N* = 10*,*596. For the list experiment, we selected a sample size to detect a small difference of 0.05 between the control list group and treatment list group. Because we framed the list questions to avoid floor and ceiling effects, we expect, on average, that the control group will agree with 2 out of the 5 items and the treatment group 2.05 of the 6 items. We selected *σ_A_* = 0*.*7 and *σ_B_* = 0*.*8, allowing the second to be larger because of the additional sensitive item. This calculation gives a sample size of *n_A_* = *n_B_* = *n_C_* = 3*,*548 and *N* = 10*,*644. To ensure our study was sufficiently powered, and to anticipate participants being lost, we recruited approximately 15*,*000 participants.

**Table S1.** Response rates for the knowledge of preventive COVID-19 behaviors by trial arm (*N* = 14,482; % represents the percentage of items correctly answered).

|  | **Do-nothing** | | | **APC** | | | **CoVideo** | | |
| --- | --- | --- | --- | --- | --- | --- | --- | --- | --- |
|  | False | True | % | False | True | % | False | True | % |
| 1. The current coronavirus can be spread by an infected person even if they look healthy | 49 | 4,859 | 99.0 | 37 | 4,740 | 99.2 | 22 | 4,775 | 99.5 |
| 2. The current coronavirus cannot be spread from person to person | 4,654 | 254 | 94.8 | 4,534 | 243 | 94.9 | 4,591 | 206 | 95.7 |
| 3. The current coronavirus cannot survive on surfaces for more than a few minutes | 4,219 | 689 | 86.0 | 4,046 | 731 | 84.7 | 4,173 | 624 | 87.0 |
| 4. Some people with COVID-19 infection may experience a cough | 55 | 4,853 | 98.9 | 43 | 4,734 | 99.1 | 38 | 4,759 | 99.2 |
| 5. Some people with COVID-19 infection do not experience a fever | 375 | 4,533 | 92.4 | 326 | 4,451 | 93.2 | 318 | 4,479 | 93.4 |
| 6. The current coronavirus spreads from person to person through small droplets from the mouth | 259 | 4,649 | 94.7 | 212 | 4,565 | 95.6 | 255 | 4,542 | 94.7 |
| 7. The current coronavirus spreads from person to person through small droplets from the nose | 644 | 4,264 | 86.9 | 598 | 4,179 | 87.5 | 617 | 4,180 | 87.1 |
| 8. You can catch COVID-19 by touching a contaminated surface and then touching your face | 122 | 4,786 | 97.5 | 80 | 4,697 | 98.3 | 58 | 4,739 | 98.8 |
| 9. Antibiotics can be used to treat COVID-19 infection | 4,370 | 538 | 89.0 | 4,332 | 445 | 90.7 | 4,343 | 454 | 90.5 |
| 10. Cleaning surfaces with soap and water is an effective way to kill the current coronavirus | 996 | 3,912 | 79.7 | 932 | 3,845 | 80.5 | 817 | 3,980 | 83.0 |
| 11. An effective way to prevent COVID-19 spread: wash your hands frequently with soap and water | 35 | 4,873 | 99.3 | 28 | 4,749 | 99.4 | 31 | 4,766 | 99.4 |
| 12. An effective way to prevent COVID-19 spread: regularly rinse your nose with salt water | 4,363 | 545 | 88.9 | 4,098 | 679 | 85.8 | 4,095 | 702 | 85.4 |
| 13. An effective way to prevent COVID-19 spread: avoid touching your face | 42 | 4,866 | 99.1 | 39 | 4,738 | 99.2 | 32 | 4,765 | 99.3 |
| 14. An effective way to prevent COVID-19 spread: avoid shaking hands with other people | 49 | 4,859 | 99.0 | 44 | 4,733 | 99.1 | 28 | 4,769 | 99.4 |
| 15. An effective way to prevent COVID-19 spread: avoid places that are crowded with people (like bars, restaurants or performances) | 47 | 4,861 | 99.0 | 39 | 4,738 | 99.2 | 25 | 4,772 | 99.5 |
| 16. An effective way to prevent COVID-19 spread: eat garlic with each meal | 4,694 | 214 | 95.6 | 4,563 | 214 | 95.5 | 4,590 | 207 | 95.7 |
| 17. An effective way to prevent COVID-19 spread: avoid sharing eating utensils with others | 207 | 4,701 | 95.8 | 191 | 4,586 | 96.0 | 148 | 4,649 | 96.9 |
| 18. An effective way to prevent COVID-19 spread: wear a face mask even if you don’t have COVID-19 symptoms | 457 | 4,451 | 90.7 | 412 | 4,365 | 91.4 | 452 | 4,345 | 90.6 |


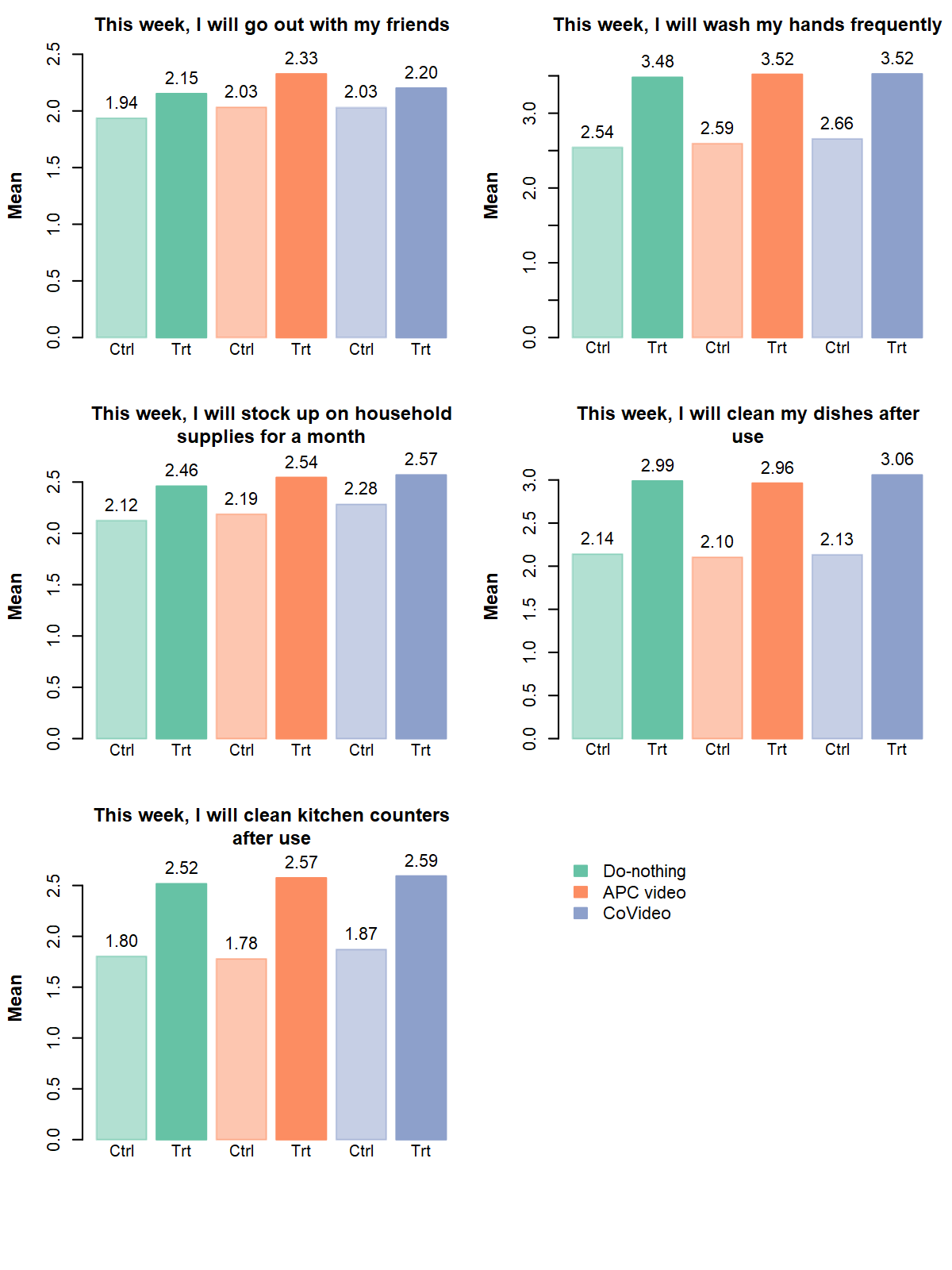


**Figure S1.** The mean scores for each of the five list experiments. Participants were randomized to a trial arm (CoVideo, attention placebo control (APC) video, or do-nothing) and list group (treatment [Trt, dark shade] or control [Ctrl, light shade]).

**Reference**

1. Rosner B. Fundamentals of Biostatistics. 7th ed. Boston, MA: Cengage Publishers; 2010. ISBN:2900538733495
